# Opportunities and Challenges for Investigating the Environment-Migration Nexus

**DOI:** 10.1007/s10745-015-9733-5

**Published:** 2015-03-29

**Authors:** Kathleen Neumann, Henk Hilderink

**Affiliations:** 1 Laboratory of Geo-information Science and Remote Sensing, Wageningen University, Wageningen, the Netherlands; 2 Department Computational Landscape Ecology, Helmholtz Centre for Environmental Research (UFZ), Leipzig, Germany; 3 PBL Netherlands Environmental Assessment Agency, Bilthoven, the Netherland

**Keywords:** Environment-migration nexus, Environmental change, Human migration, Research methodologies

## Abstract

Environmental change is an acknowledged factor influencing human migration. Analytical research regarding the relationship between the environment and human migration has increased in recent years yet still faces numerous hurdles, partly due to limited availability of suitable data. We review available data and methodologies for investigating the environment-migration nexus, identifying data inconsistencies resulting from the combination of different sources and illustrating the underlying reasons for them. We discuss a number of methods for investigating the environment-migration relationship, including frameworks and concepts; surveys; empirical, quantitative methods; and simulation approaches. Based on this overview, we offer recommendations for improved analyses of the environment-migration nexus including reporting data inconsistencies and uncertainties, combining approaches and data sources, and developing multiple-study approaches.

## Introduction

Human migration has been studied extensively in recent decades. However, only recently has a paradigm shift arisen in favour of acknowledging the complex contribution of environmental change in migration processes (Black *et al*. 2011; McLeman and Smit 2006; Piguet 2012; Piguet *et al*. 2011). Recently, scholars have increasingly recognized that—with the exception of extreme conditions—environmental change alone does not cause migration. Rather, it is the interaction of environmental factors with non-environmental factors that account for the causes of migration (Black 2001; Castles and Miller 1998; Parnell and Walawege 2011). However, little is known regarding the importance of environmental factors in relation to other factors and the degree to which these factors interact. In fact, compelling empirical studies regarding environment-migration relationships have emerged only recently, and consequently their number remains rather low. The principal obstacles to these studies include a rather limited understanding of the interactions of environmental and non-environmental factors that influence migration and a general lack of sufficient data covering both environmental factors and migration within a given place and time (Bilsborrow and Henry 2012). Nevertheless, various datasets and methods have been used to investigate environment-migration relationships (McLeman 2013; Piguet 2010) originating from a variety of scientific fields and sources, each with its own strengths and/or limitations, which can be enhanced and/or overcome by combining methods, although this approach has rarely been used. Further, many environment-migration studies do not explicitly address the quality and applicability of the data and/or methods utilised. However, dealing with different spatial and temporal scales, definitions, methods, and data can yield a variety of empirically drawn conclusions regarding the relationship between the environment and migration. For example, Beine and Parsons (2012) conclude that climate change did not drive international migration during the second half of the twentieth century at the global scale, whereas Marchiori *et al.* (2012) conclude that in the same period, temperature and rainfall anomalies were responsible for the migration of at least 5,000,000 people in sub-Saharan Africa. Based on their own research, Reuveny and Moore (2009) argued that weather-related natural disasters and cumulative environmental degradation play a role in migration; however, this conclusion was not confirmed by Naudé (2010) with respect to sub-Saharan Africa. Based on a meta-analysis, Lilleør and Van den Broeck (2011) conclude that climate change can have a variety of effects on migration in less-developed countries. Although many studies included in the meta-analysis have provided evidence to support a negative relationship between rainfall and migration, the authors highlight the challenge of identifying the precise mechanisms by which rainfall influences migration; but clearly, agricultural production and food availability play a central role. Thus, the currently available literature provides differing conclusions regarding the environment-migration nexus, and it remains unclear whether these differences are related to methods and/or data or contextual differences.

This paper reviews both the data and methods used in environmental migration studies with the aim of identifying critical issues related to their usefulness in assessing the environment-migration nexus. We do not seek here to analyse the interaction between environmental and non-environmental factors contributing to migration. We first discuss the object of study for the environment-migration nexus. Second, we provide an overview of data sources from the environment and population domain and identify inconsistencies related to the use of these data. Third, based on the data overview and the associated consistency problems, we discuss approaches for analysing environment-migration relationships. Finally, we provide a series of recommendations for improving the analysis of the environment-migration nexus.

## The two Components of the Environment-Migration Nexus

There are two key components of the environment-migration nexus: the natural environment and its changes; and migration as a possible response strategy to environmental change.

Environmental changes can occur suddenly (*fast-onset* changes) in the form of a flood, hurricane, tsunami, or earthquake, or they can occur gradually (*slow-onset* changes), for example in the case of climate change, land degradation, and rising sea level. In either case environmental changes may trigger migration. The environment-migration nexus is generally better understood for *fast-onset* changes, as well-illustrated by the relatively large number of scientific publications regarding migration due to Hurricane Katrina (a *fast-onset* change) in 2005 (Elliott and Pais 2006; Fussell *et al.* 2010; Myers *et al*. 2008). There are several reasons for this difference. Usually, *slow-onset* changes are more complex in nature than *fast-onset* changes, limiting our knowledge regarding the interactions and mechanisms that cause migration. Moreover, data related to *fast-onset* changes are more accessible than *slow-onset* change data, as the latter require consistent time series, which are generally rare.

There are many types of migration of interest for studying the environment-migration nexus, including internal versus international migration, rural-rural versus rural–urban migration, and temporal versus permanent migration. Internal migration occurs within a given country by crossing sub-national boundaries (for example, by moving between districts or provinces), whereas international migration involves crossing national borders. The origins and destinations of migrants are particularly interesting for studying the causes and consequences of migration including potential associated tele-connections. Generally speaking, perceived or objective differences in living conditions, including the natural environment, between the place of origin (*push* factors) and the destination (*pull* factors) can lead to migration. In contrast, the temporal dimension of migration is typically more difficult to determine. Usually, migrants are reported as persons who migrated 5 or 10 years ago. However, based on this information alone, no conclusions can be drawn with respect to whether the migration was temporary or permanent, as their return migration can occur after 5–10 years. In addition, a migrant’s decision to move either temporarily or permanently can change at any point in time after the migration event, which makes reporting this migration type difficult. Moreover, to study migration in response to *slow-onset* changes, periods longer than a few years must be taken into account, given the long response time of these changes.

## Overview of Environmental and Migration Data

Here, we focus on a few environmental factors that are widely acknowledged as potentially having an influence on migration, including land cover and land use, land degradation, and climate (variability).

### Land Cover and Land Use-Related Data

A wide variety of data sources that use remote sensing to detect land cover are available, with aerial photography and satellites being the most common. There are several remote sensing techniques, all based on the principle that different types of surfaces reflect and absorb different frequencies of solar radiation. Therefore, the reflection of solar radiation depends on the land cover, which does not necessarily equal the land *use*, although obtaining land use information using land cover data is possible to some extent (Verburg *et al.* 2010). Remote sensing time series can be used to investigate changes in land cover that can be valuable for studying environment-migration relationships, particularly with respect to natural vegetation types and cropping. Several instruments can provide consistent land cover time series, including (in order of increasing spatial resolution) AVHRR, MODIS, Spot, Landsat, Quickbird, and Ikonos. Surveys and censuses are also valuable sources of land use data, since unlike remote sensing, they explicitly report land use and land use management rather than land cover. However, although agricultural censuses provide information regarding land management and resource use focusing on administrative units, including labour, irrigation, fertiliser application rates, and crop yields, most do not include information regarding natural and semi-natural land use types (Verburg *et al.* 2010). Information regarding land tenure and property rights can best be obtained from cadastres. In particular, information regarding common property resources can be valuable for investigating environment-migration relationships, as these are usually used first as land becomes scarce, often causing land degradation.

### Land Degradation Data

Land degradation data are usually derived from remote sensing or expert judgement (Table 1). The Global Assessment of Soil Degradation (GLASOD) database contains information regarding the type, extent, degree, rate, and principal causes of degradation for the entire globe (Oldeman and Van Lynden 1996; Oldeman *et al.* 1991). The primary criticism of GLASOD has been its subjectivity, which has hindered coherent and consistent comparisons of soil degradation levels among locations. In the past decade, land degradation datasets derived from remote sensing data have been developed that, in addition to being objective, also account for the temporal dimension of land degradation. The Global Inventory Modeling and Mapping Studies (GIMMS) dataset is a Normalised Difference Vegetation Index (NDVI) spanning the period from 1981 through 2006 (Tucker *et al*. 2004, 2005). The dataset, created using images from the AVHRR sensor, indicates where and to what extent land degradation—measured as a disturbance-driven long-term loss of net primary productivity (NPP) from which the land cannot recover without assistance (Bai *et al.* 2008)—either increased or decreased. One obvious disadvantage of using land productivity data is their dependence on precipitation—a decline in precipitation usually causes a decline in land productivity, which is reflected in a decreasing NDVI trend that does not necessarily indicate land degradation. To address this problem, Bai *et al.* (2012) developed an approach to identify changes in annual biomass production by disentangling the likely impact of climate factors—including temperature and precipitation—from other factors that can influence the NDVI. In addition to changes in NPP and NDVI, rain use efficiency—defined as land production per unit rainfall—can serve as a suitable proxy for land degradation in regions in which rainfall is the most limiting factor on NPP, such as drylands (Illius and O’connor 1999; Le Houerou 1984). Global rain use efficiency data are available from the Land Degradation Assessment in Drylands (LADA) project (http://www.fao.org/nr/lada). For regional- and national-level land degradation assessments for Senegal, Cuba, Argentina, China, and Tunisia, LADA combined several quantitative data sources with the help of regional expert knowledge (Biancalani *et al.* 2011).

**Table 1:** Overview of the thematic, spatial and temporal properties of various global land degradation data

Dataset	Thematic properties	Unit of measure	Spatial resolution	Temporal extent
GLASOD	Expert-based judgement of human-induced soil degradation	Degree and extent of degradation severity [ordinal scale]	Mapping units at the scale of 1:10 million	Situation around 1990
GIMMS	Normalised Difference Vegetation Index (NDVI)	Change in net primary productivity [kgC/ha/year]	5 arc minutes	1981–2006
Bai *et al.* (2012)	Changes in biomass production due to 1) climate related factors, and 2) non-climate related factors	Change in net primary productivity [%/year]	5 arc minutes	1981–2006
LADA	Biomass production per unit rainfall	Change in rain use efficiency [sum NDVI/sum precipitation/year]	5 arc minutes	1981 to 2003

Additional terrestrial data include (point) measurements of stream gauges, coastal erosion, and geotectonic conditions, e.g., earthquake faults and subsistence rates for coastal and atoll states. Typically, such data cover sub-national scales and are provided by (sub-) national authorities.

### Climate Data

From the wide variety of climate databases we focus here on (global) climate data that can be used —at least in general—to investigate the relationship between climate change and migration. Climate data such as precipitation, temperature, and evapotranspiration are usually obtained from local weather stations that record hourly weather information. Climate information from several weather stations is often combined using interpolation techniques in order to derive data for larger areas that can reach global coverage. Examples of this approach include the Worldclim database, which contains select climate variables at 1-km grid cell resolution averaged over the period from 1950 through 2000 (Hijmans *et al.* 2005). In addition, the CRU-TS 3, ERA-interim, and NCEP-DOE databases provide climate time series for various periods in the past (Dee *et al.* 2011; Kanamitsu *et al.* 2002; Mitchell and Jones 2005). Such time series are essential for evaluating spatio-temporal patterns of climate change that are increasingly considered to be a driver of migration (Foresight 2011a; IOM 2008). The primary drawback of global time series is their relatively low spatial resolution (Table 2), which may be insufficient for assessing the relationship between climate change and migration at the regional or local scale. Moreover, interpolation techniques may result in data errors for regions with a low density of weather stations, and these can be particularly problematic when attempting to assess the socioeconomic effects of climate change (Auffhammer *et al.* 2013). This is particularly critical if error estimates for individual grid cells are not provided, as in the case of the CRU data. The UDEL dataset (Willmott *et al.* 2010) addresses this problem, at least in part, by providing grid cell-specific error estimates based on the spatially interpolated value of cross-validated absolute errors at nearby stations (Lobell 2013).

**Table 2:** Overview of the thematic, spatial, and temporal properties of various gridded global weather and climate data

Dataset	Variables	Spatial resolution	Temporal extent	Temporal resolution
Worldclim	Monthly minimum, maximum and mean temperature, monthly total precipitation	1 km	1950–2000	One average value for 1950–2000
CRU	Cloud cover, diurnal temperature range, frost day frequency, precipitation sum, daily mean temperature, average daily minimum and maximum temperature, vapour pressure, wet day frequency	0.5°	1901–2013	Monthly
UDEL	Average temperature, total precipitation, terrestrial water budgets, moisture indices	0.5°	1900–2010	Monthly
ERA-interim	Minimum and maximum temperature, precipitation, radiation, relative humidity	1.5°	1979–present	Daily and every 3 h
NCEP-DOE	Minimum and maximum temperature, precipitation, radiation, relative humidity	2.5°	1979–2014	Daily
NASA-Power	Minimum and maximum temperature, precipitation, radiation, relative humidity	1°	1983–present (except precipitation: 1997–present)	Daily
NASA-Merra	Minimum and maximum temperature, precipitation, radiation, relative humidity	1/2 × 2/3°	1997–present	Daily and hourly
MODIS	Drought Severity Index (DSI)	0.5°	2000–2011	Annual and every 8 days

In addition to weather stations, satellite data are a valuable source of climate information, particularly with respect to natural hazards. Remote sensing methods have improved significantly in recent decades, and combining these methods with modelling techniques has enabled us to understand better where and when a particular natural hazard is most likely to occur. Relevant, detectable information can include rainfall intensity, river height and weight, and soil moisture (Tralli *et al*. 2005). Further, at the regional scale thermal properties detected by satellites such as NOAA AVHRR and MODIS have been used to derive indices such as the Temperature Condition Index (TCI) (Singh *et al*. 2003) and the Drought Severity Index (DSI) for monitoring droughts (Wan *et al*. 2004).

Besides the gridded data special data sources exist for disasters, including the databases EM-DAT (http://www.emdat.be/) and DesInventar (www.desinventar.net) that provide information regarding various types of disasters, including natural disasters, number of people affected and estimates of economic damages at the regional or country level.

### Migration Data

Despite a wide variety of available demographic data, consistent and reliable migration data—particularly with respect to environmental change—is somewhat limited (Table 3).

**Table 3:** Overview of the thematic, spatial, and temporal properties of various sources of international migration data

Dataset	Variables	Spatial resolution	Spatial extent	Temporal extent	Temporal resolution
World Population prospects	International net-migration flows	Country	250 countries and territories	1951–present	Decade
Global Migrant Origin Database	International bilateral migration stocks	Country	226 countries and territories	2000	For 2000 only
Özden database	International bilateral migration stocks	Country	226 countries and territories	1960–2000	Decade
Eurostat	Determinants and stocks of international bilateral migration	Region	Turkey, Morocco, Egypt, Ghana, Senegal	For the beginning of the 1990s	For the beginning of the 1990s only
DIOC-OECD	Immigrants in OECD countries	Country	All OECD countries except Iceland	2000	For 2000 only
Ortega and Peri database	Immigrants in OECD countries	Country	15 OECD countries, 120 countries of origin	1980–2006	Yearly
CIESIN	Net-migration	30 arc-seconds	global	1970–2010	Decade

At the global level, the UN Population Division biannually reports historical demographic data as well as future projections for nearly 250 countries (United Nations 2010), including information regarding net migration, which is based on a combination of observations and model derivations. Country-specific migration is analysed using a pooled approach in which no specification is given with respect to origin and destination, although the global net sum equals zero. An advantage of this database is its complete global coverage and use of a consistent methodology to derive migration flows. However, it provides only net migration information, without specifying in-migration and/or out-migration, places of origin and/or destination, or the age structure of the migrants. A similar approach was applied by CIESIN, which obtained migration flows from population changes and applied those flows to a global grid at 30 arc-second resolution (Foresight 2011b; Sherbinin *et al.* 2012) by combining various data sources such as surveys and national statistics. Grid cell-specific birth and death rates were obtained using a downscaling procedure and combined with population density numbers to acquire net-migration density per grid cell. The Global Migrant Origin Database reports international bilateral migration stocks for 226 countries around the year 2000. Although this database is unique, it contains large data gaps and covers only international migrants (Parsons *et al.* 2007). Another limitation with respect to analysing the environment-migration nexus is that it specifies neither migration flows nor the underlying causes of the migration. More recently, Özden *et al.* (2011) extended the Global Migrant Origin Database with their report of international bilateral migration stocks for 226 countries, corresponding to the last five completed census rounds from 1960 through 2000.

In addition to global databases, there are databases that cover both stocks and (annual) flows of migrants based on their origin and destination for developed countries, including the European Union (Eurostat 2000) and the OECD member countries (OECD 2008; Ortega and Peri 2013). In addition, Eurostat (2000) commissioned a survey that focussed on the motives of migrants—including environmental factors—in Turkey and four African sending countries with respect to two European receiving countries.

At the national level, demographic surveys report national data regarding population structure, education, employment, religion, ethnicity, and (occasionally) migration. However, information with respect to migration is usually limited to place of residence for *n* years (e.g., 5 or 10 years) prior to the survey, and the reasons underlying the migration are usually not surveyed. Interviewing individuals from the non-migrant population (including family members of migrants) may shed light on timing and motives of migrants, though such information tends to be incomplete and biased. If migration data are not available, proxies may be used, e.g., van der Geest’s (2011a) work in Ghana.

The household level is likely the most appropriate level for obtaining information regarding the causes of migration, including environmental change. For example, the Ethiopian Rural Household Survey (IFPRI 2011) and the Indonesian Family Life Survey (available online at http://www.rand.org/labor/FLS) provide longitudinal household-level information that covers migration issues. As part of the Africa Migration Project (Plaza *et al.* 2011) over 70 migration and remittance household surveys were conducted from 1990 through 2006 in Burkina Faso, Kenya, Nigeria, Senegal, South Africa, and Uganda, including data on the motives of migrants. As with many surveys, the primary limitations were sample coverage (i.e., too little) and representativeness of sampled households (i.e., different spatial levels of statistical representativeness). Surveys, such as the Displaced New Orleans Residents Pilot Survey (DNORPS) followed by the Displaced New Orleans Residents Survey (DNORS), conducted after Hurricane Katrina (Fussell *et al*. 2010; Sastry 2009; Sastry and Peterson 2010), which are concerned with people displaced by (natural) hazards, collect data on new locations, status, and well-being of the displaced residents,

## Consistency Issues Related to Combining Environmental and Migration Data

Analysis of environment-migration relationships is often hampered by inconsistencies in the data, e.g., with respect to time, themes, and space, which preclude both comparison and integration of various data sources.

To properly assess the relationship between environmental change and migration, the data must have the appropriate temporal resolution. For example, if migration is related to a season, such as rural–urban migration during the dry-season in West Africa, time-series data regarding climate and migration must be at the monthly or quarterly level rather than annual (Kniveton *et al.* 2008). However, whereas climate time series usually have high temporal resolution (e.g., on the order of days or months), migration data are generally available only at a coarser temporal resolution. Thus, Kniveton *et al.* (2008) concluded that a change in climate at time *t* cannot be used to explain migration at time *t* + 1, as such high-resolution data regarding migration are generally not available. Consistent long-term data are essential for monitoring and analysing the dynamics of the environment-migration relationship. Ideally, these data would be derived from the same data source and would be based on the same methods. Although this approach is generally feasible for climate time series, it usually cannot be used for other environmental data, including land cover and land degradation. For example, mapping based on remote sensing data is usually funded in the short-term and tailored to the specific information requirements of the funding agencies. Consequently, a wide variety of remote sensing data products exist that are based on different processing and interpretation techniques, which hampers comparability and reduces consistency among datasets (Verburg *et al.* 2010). Data obtained from surveys can also contain inconsistencies. As discussed above, demographic surveys are infrequent and can be subject to changes between surveys with respect to sampling methods, survey questions, classification systems, and definitions that can affect the measurement of migration (Nowok *et al.* 2006). Such differences can arise from reporting differences either between countries or between years and lead to thematic inconsistencies that can hamper cross-national or cross-regional comparisons of migration patterns (e.g., Beer *et al.* 2010).

The spatial resolution of global environmental data is often sufficient for analyses performed at the regional, national, and global scales. However, relatively coarse resolution (i.e., large pixel size) can obscure spatial variability in environmental conditions, including the occurrence of linear or small landscape elements at the local level. This results in a mismatch with respect to spatial detail between environmental and migration data if the latter is provided at higher spatial detail (e.g., at the household level). In turn, migration data at the province level are likely too coarse for investigating their relationship with local environmental changes such as flooding and soil degradation. Although aggregating data to the same level of analysis can overcome this problem, it can also introduce errors. Indeed, the inadequate or inappropriate aggregation of data is another potential source of spatial inconsistencies. Both the level and the method of aggregation can largely determine the resulting data structure and ultimately results of the analyses. Although the impact of applying various aggregation methods and various spatial resolutions has been studied extensively in the environmental domain (Dendoncker *et al.* 2008; Ershadi *et al.* 2013; Folberth *et al*. 2012; Kok and Veldkamp 2001; Trivedi *et al.* 2008; Verburg *et al*. 2010), it has drawn surprisingly little attention with respect to investigating environment-migration relationships.

## Research Methodologies for Analysing the Environment-Migration Nexus

In this section, we discuss several concepts and methods for investigating environment-migration relationships, keeping the aforementioned inconsistencies in mind to make optimal use of the available data.

### Framework and concepts

The tight relationships between environmental and non-environmental factors that drive migration make investigating “pure” environment-induced migration difficult and even possibly inappropriate. The complex interplay among the various driving factors has been acknowledged in several recent frameworks (Black *et al.* 2011; McLeman and Smit 2006; Meze-Hausken 2000; Morrissey 2013; Perch-Nielsen *et al*. 2008). For example, Black *et al.* (2011) elaborate how drivers of migration in general can be affected by environmental change, noting that environmental change can be either a direct or an indirect driver of migration and emphasising the need for placing environment-migration interaction in a broader socioeconomic context. Building upon this work, Neumann *et al.* (2015) conceptualized environmental drivers of migration in drylands and applied a cluster analysis for analyzing and mapping these drivers at the global scale.

Some scholars use the concept of vulnerability as a starting point for understanding environment-induced migration. For example, McLeman and Smit (2006) developed a conceptual climate change-migration model to illustrate how household capital affects adaptive capacity and—consequently—the decision to migrate. Similarly, Meze-Hausken (2000) determined the adaptive capacity of farmers in African drylands using a combination of socioeconomic and environmental factors, including livestock keeping and non-agricultural income and/or remittances. Using a model in which exposure to climate change is expressed as both short-term and long-term changes in rainfall variability, which potentially restricts water and food availability, she illustrated the vicious cycle in which increasing vulnerability to climate change causes a decline in adaptation options, which consequently increases vulnerability even further. Perch-Nielsen *et al.* (2008) reviewed a number of case studies for expanding the “common sense” link between floods and rising sea level on one hand, and vulnerability and migration on the other to create a conceptual model that includes a wide range of intervening variables. In addition to these studies, other scholars have investigated environment-induced migration in the context of vulnerability by exploring the interactions between environmental change and migration using descriptive methods (McLeman and Hunter 2010; Warner *et al.* 2010). A valuable approach for understanding responses of households to external vulnerabilities is the Sustainable Livelihoods Approach (SLA), which addresses the vulnerability of livelihoods to shocks and stress as well as strategies for coping with and recovering from them (Chambers and Conway 1991), including migration (Kniveton *et al.* 2009).

Another approach for investigating the environment-migration relationship is the concept of social and/or ecological resilience. Whereas vulnerability focusses on power and the limitations of an individual agency, social resilience is the ability of groups or individuals to cope with external stresses and disturbances, including environmental change (Adger 2000), and can be increased through social relationships. Ecological resilience is the ability of an ecosystem to respond to disturbances through damage resistance and rapid recovery. Adger (2000) and Adger *et al.* (2002) note that migration is a key indicator of social resilience, but they also stress that the presence or absence of migration in a given area is insufficient to draw conclusions regarding social resilience. Deshingkar (2012) investigated the causal links between environmental change and migration, remittances, and resilience in Western Mexico, the Central Plateau of Burkina Faso, and Jharkhand in Eastern India, and found that remittances from out-migrants potentially improve the resilience of income-poor households to external shocks. However, whether declining ecological resilience can trigger further out-migration remains to be determined. Central to the concepts of vulnerability and resilience is the issue of cumulative causality, which—in the case of migration—can be considered a threshold at which environmental impacts are so severe (or so frequent) that in situ adaptation options are insufficient and people migrate (Bardsley and Hugo 2010). However, current conceptual models accounting for such thresholds are rare and challenged by the enormous complexity of human-environmental interactions (Meze-Hausken 2008).

### Surveys

Bilsborrow and Henry (2012) suggest three approaches for using household surveys to study the environment-migration relationship at the local level: 1) Combine existing population data from various sources, 2) Design a new survey, and 3) Extend an on-going or planned survey that was originally developed for another purpose. The first approach is exemplified by combining province-level population data with household-level event-history data (Henry *et al.* 2003) to improve understanding of the relationship between rainfall and migration in Burkina Faso. They conclude first, people from more arid regions are more likely to migrate than people from wetter regions, and second, short-term rainfall deficits tend to increase rural-rural migration and decrease short-term migration to distant destinations (Bilsborrow and Henry 2012). Similarly, Dillon *et al.* (2011) combined population data, including household characteristics, from the 1988–1989 Northern Nigerian Household Survey with data—for the same households—from a tracking survey conducted in 2008 regarding changes in place of residence and reasons for migration. This yielded a dataset that was used to investigate to what extent Nigerian households migrate in order to avoid temperature-related agricultural risks. Probably the major challenge in merging data arises from spatial and temporal inconsistencies among various sources. Tracking surveys (or longitudinal surveys) use repeated observations of (migrant) households or individuals over time and are a major tool in improving assessment of migration-related changes in livelihood. They are also generally less limited than single retrospective surveys with respect to respondents’ recall of their migration events. Beegle *et al.* (2010) used a tracking survey to investigate migration in Tanzania from 1991 through 2004. They found that without tracking first round interviewees, the percentage of second round interviewees would have decreased from 82 to 52 %.

Design of a new survey to collect both migration and environmental data as an alternative to using existing surveys has yielded promising results in several studies (e.g., Barbieri and Carr 2005; Gray and Bilsborrow 2013; Meze-Hausken 2000; Paul 2005; van der Geest 2011b). An obvious advantage is the opportunity to collect data that capture multiple dimensions of the environment-migration nexus to avoid gaps in analyses that can occur in existing surveys. On the other hand, the disadvantages include time and costs of preparing the survey and conducting—and in case of longitudinal surveys, replicating—interviews, which can be significant.

Lastly, an on-going or planned survey originally developed for another purpose can be adapted by incorporating additional questions. For example, Bilsborrow and Henry (2012) added questions regarding within-department migration and land use to the standard Demographic Health Survey (DHS) of Guatemala in order to explore the link between in-migration and deforestation in the region of Peten. The primary challenges in extending an existing survey include coping with possible resistance by interviewees (given the different nature of the extended survey) and avoiding a survey too long to be practical.

Regardless their utility, household surveys are limited in their ability to assess the extent that environmental change is a contributing factor to migration in so far as informants are likely to have difficulties disentangling climate variability from other migration motives (Kniveton *et al.* 2008). This problem may be addressed by first identifying respondents’ motives for migrating and subsequently testing those for sensitivity to climate change.

### Empirical, Quantitative Analyses

Of the variety of statistical methods available, correlation analysis is a simple method for assessing the nature of the relationship between environmental conditions and migration. For example, van der Geest *et al.* (2010) combined the 2000 Ghanaian census data with GIMMS and CRU data and calculated the correlations between migration, NDVI, and precipitation at the district level in Ghana. Their findings suggest that out-migration occurs primarily in arid districts, whereas in-migration is more common in humid districts. A second—albeit rather counter-intuitive—finding is that districts with more out-migration tend to have a more positive NDVI than districts with more in-migration, and this difference may be related to the recovery of natural vegetation after the population pressure has declined. It is important to note that temporal changes in both NDVI and precipitation were accounted for (rather than observations at a single point in time). However, the correlation coefficients were relatively weak, which suggests two things. First, other (non-investigated) factors, including agricultural production and employment opportunities, are likely to play an important role in migration. Second, the indicators that were used may have been inadequate. For example, changes in seasonal rainfall or changes in rainfall variability may be a more suitable rainfall-related indicator of migration than changes in annual rainfall.

Regression analysis can be used to investigate the association between a dependent variable (e.g., migration) and one or more independent variables (e.g., soil fertility, land degradation, aridity, etc.). This approach is also referred to as predicting the value of the dependent variable. As Kniveton *et al.* (2008) note, the accuracy of the prediction is dependent on the strength of the relationship between the variables, the nature of the relationship (e.g., linear versus non-linear), and the ability to gather reliable contextual information to properly capture the drivers of migration. Regression analyses have resulted in promising results for several studies, including regions in Ecuador (Gray and Bilsborrow 2013), Mexico (Hunter *et al*. 2013), South Africa (Leyk *et al*. 2012), Kenya and Uganda (Gray 2011), Bangladesh (Joarder and Miller 2013), as well as in the US (Gutmann *et al.* 2005). For Mexico, Feng *et al.* (2010) used regression analyses to investigate the state-level impact of climate variability on maize yield and subsequent out-migration to the United States. Based on historical data, their findings suggest that a 10 % decrease in maize yield would cause a 2 % increase in out-migration. The applied approach has initiated a methodological controversy between Auffhammer and Vincent (2012) and Feng and Oppenheimer (2012), with the former contesting this causal relationship based on their own statistical estimations. Henry *et al.* (2003) used generalised linear modelling, a flexible form of linear regression, to explore the role of environmental factors in inter-provincial migration in Burkina Faso. Using a step-wise approach, the authors included socio-demographic, climate, and land degradation variables in their regression models to identify each variable’s individual explanatory power. They concluded that socio-demographic variables have higher explanatory power than environmental variables. Interestingly, in their analysis of out-migration flows from the more arid northern provinces, environmental variables had higher explanatory power than the socio-demographic variables, in contrast with the results obtained for the southern provinces, which have a more favourable climate. Although it may be challenging to clearly separate socioeconomic from environmental factors, such a step-wise approach can potentially help comparing the contributions of environmental factors and non-environmental factors.

Data related to human-environmental systems often have a hierarchical spatial (e.g., municipalities, provinces, countries), or thematic (e.g., individual, household, village) structure. For example, data regarding migration are available at the district level, data regarding soil conditions are available at the field level, and climate data are available at the grid level. Hierarchically structured data statistically analysed at multiple levels can better account for statistical within-level and between-level relationships, thus avoiding misleading results (Snijders and Bosker 1999). Nawrotzki *et al.* (2013) applied multi-level analyses to assess the impact of rainfall changes on migration from Mexico to the US using cross-sectional data for 2000. This, however, cannot account for possibly time-lagged migratory responses. To account for such delayed responses, Henry *et al.* (2004) performed discrete-time event history analyses to investigate the impact of both average rainfall and rainfall variability on out-migration in Burkina Faso. They combined individual-level demographic variables and community-level information regarding land availability and infrastructure with rainfall data to explain the determinants of migration from rural areas from 1960 through 1998. They concluded that people from more arid regions are more likely to migrate than people from more humid areas. Importantly, they found that even short-term deficits in rainfall are likely to increase long-term rural-rural migration (see also Gray 2009; Massey *et al.* 2010, and Gray and Müller 2012a,b for the Ecuadorian Andes, Nepal, Ethiopia, and Bangladesh respectively).

In contrast, Ezra and Kiros (2001) combined individual, household, and community factors to explain out-migration in drought-prone rural areas of Ethiopia. They examined how a wide variety of environmental and economic factors can explain migration in areas under environmental pressure, rather than the relationship between droughts and migration per se. This approach is particularly valuable for gaining a more general understanding of migration drivers within the context of environmental stress. Nevertheless, these approaches also have drawbacks that bear mentioning. First, while statistical analyses can provide important insights into cause-and-effect relationships between environmental factors and migration, which would not be available from qualitative approaches alone, the conclusions also depend on the analyst’s judgement. For example, this can be relevant when assessing the relationship between migration and land degradation, with the latter serving as a potential cause *and* consequence of migration. Second, statistical analyses are usually based upon pre-defined spatial units for which the migration information is provided. If the units are not sufficiently detailed, they may fail to adequately represent the spatial variability of the environmental factors, and as a consequence, the statistical relationships can be underestimated. For example, migration data provided at the province level may be too coarse to allow for investigation of their relationship with flooding and/or soil degradation that occurred on a smaller scale. Multi-level analyses can help overcoming this shortcoming, but only if the statistical units are defined appropriately.

### Simulating Migration

Simulation of future trajectories of environment-induced migration remains in its infancy and a relatively limited number of promising modelling studies have been conducted to date. McLeman (2013) argues that current modelling efforts are based only on general assumptions with respect to migration behaviour, which limits their applicability in predicting future migration trends. Moreover, no integrated modelling approaches that can consider a wide range of possible migration strategies in addition to other adaptation strategies have yet been developed. The major challenges faced by current modelling efforts include a rather limited understanding of the environmental and non-environmental drivers of migration (including their interactions), as well as a lack of reliable data.

It should be emphasised that simulation studies such as those discussed here are generally based on empirically established statistical relationships, which makes them partially overlapping with the quantitative analyses reviewed above. Agent-based modelling (ABM) incorporates known migration behaviour of individuals (i.e., agents) into projections of future behaviour based on hypotheses captured in scenarios. The strength of this approach lies in its ability to capture the broad spectrum of migration behaviours among individuals. Although ABM has been widely used in migration studies, few researchers have used it to study the environment-migration nexus (Piguet 2010). Kniveton *et al.* (2011) used ABM to investigate the role of the environment in an individual’s decision to migrate in Burkina Faso. In addition to projections regarding climate change, they included a range of economic, social, demographic, and political factors in their scenarios. Their findings suggest that the total migration flux will increase approximately four-fold from 2010 to 2060. Interestingly, the largest migration fluxes are projected to occur in a scenario in which dry climate is combined with low demographic growth and local social and political governance. Moreover, their findings with respect to international migration fluxes contradict the results of Henry *et al.’s* (2004) earlier study. Similarly, Hassani-Mahmooei and Parris (2012) used ABM to simulate migration as a consequence of climate change in Bangladesh, considering a range of economic, social, demographic, and environmental factors in their scenarios. Their results indicate that through the year 2050, 3–10 million people will likely migrate internally, with the most regions of origin being the drought-prone western districts and the southern areas, which are prone to cyclones and floods.Piguet (2010) argues that the limited use of ABM in the field of environment-induced migration compared to other fields has two possible explanations. First, available knowledge regarding how individuals react to environmental change is still insufficient for deriving reliable behaviour-based modelling rules. Second, the variety of potential environment-related stimuli that drive migration may not be sufficiently represented by the existing modelling rules.

At an aggregated level, alternate modelling techniques have been used to quantitatively explore possible trajectories of environmental change and human migration. For instance, Feng *et al.* (2010) used results from regression analyses to simulate the effect of climate variability on maize yield in Mexico and subsequent out-migration to the United States. Based on four different scenarios that incorporated climate change and adaptation, the authors concluded that 2-10 % of the current Mexican population migrate by 2080 in response to a climate change-related decline in agricultural production. In an integrated assessment model based on a previous study by Döll and Krol (2002), Krol and Bronstert (2007) simulated the relationship between expected climate change, water availability, agricultural economy, and migration for municipalities in northeast Brazil. They found that migration is driven primarily by mean municipal income, which is a function of climate change, water availability, and agricultural yields. In turn, population changes, including migration, drive water availability and the agro-economy. Hence, the feedback pathways between climate change, agriculture, and migration are continuously updated in the model (see also Feng *et al.* 2010). In contrast, Barbieri *et al.* (2010) simulated the impact of climate change on agricultural production and consequently migration for the same region by estimating state-level net-migration rates for three scenarios. Interestingly, net migration in northeast Brazil is negative in all scenarios for all periods; hence, out-migration is projected for all scenarios, although the absolute magnitude of the population decline varies among the scenarios.

Marchiori and Schumacher (2011) used an econometric approach in order to project global international migration in 2050 as a consequence of global temperature increases due to economic development. Their approach was based on the simple assumption that migration is driven by regional-level differences in welfare. As a result, they concluded that a decline in productivity in the Southern Hemisphere will increase the number of migrants four-fold. Their study sheds light on the inter-connections among climate change, economic development, and immigration policies; however, their approach is relatively abstract by roughly distinguishing between the Northern and Southern Hemispheres.

What is evident from these simulation studies is that they all account for the feedback between environmental factors and economic development rather than projecting migration as a straightforward consequence of environmental change. Unlike the ABM studies, the studies by Döll and Krol (2002), Krol and Bronstert (2007), Barbieri *et al.* (2010), Feng *et al.* (2010), and Marchiori and Schumacher (2011) are based on broad, regional-level assumptions regarding migration decisions. Nevertheless, these studies clearly represent a valuable contribution to the exploration of possible future trajectories of climate-induced migration.

## Conclusions and Recommendations for Analysing the Environment-Migration Nexus

We conclude by proposing recommendations for advancing the investigation of the environment-migration nexus and therefore our understanding of this interaction.

Although uncertainty analyses are central in many environmental change assessments, to date they have drawn relatively little attention in studies investigating the environment-migration nexus. Gemenne (2011) stresses that existing predictions and estimates of environmental migrants are highly controversial, as many lack sound documentation of underlying assumptions, uncertainties, and/or potential errors. Uncertainties and errors can be related to the assessment of environmental change processes, migration flows, the examined linkage between environmental change and migration behaviour, or the respective data. Uncertainty assessments remain particularly underreported in qualitative and simulation studies, despite availability of theoretical frameworks for systematic uncertainty analysis, especially for model-based approaches (Walker *et al.* 2003). Indeed, quantitative simulation studies can benefit from a variety of quantitative uncertainty assessments, including sensitivity analysis, error propagation equations, inverse modelling, scenario analysis, Monte Carlo simulation or Bayesian statistical modelling (Refsgaard *et al.* 2007). In fact, Bayesian approaches were also applied for interpreting uncertainties in qualitative modelling studies, for example qualitative network analyses (Melbourne-Thomas *et al.* 2012). Although these approaches have been widely acknowledged in environmental studies they have not yet conquered the field of simulating environment-induced migration, which is likely related to the fact that simulating those processes is a rather new endeavour (McLeman 2013). Clearly, uncertainties related to data and/or methods can lead to underestimation or overestimation of migration that is driven by environmental change. Scholars must be cognisant of inconsistencies and uncertainties in environmental and migration data when studying their impact on the results of their analyses, and these factors should in turn be communicated openly and accurately.

Analysing the causes of inconsistencies and errors is a positive step towards obtaining more appropriate data for investigating the environment-migration relationship. Data inconsistencies can also indicate the existence of complementary information that—at least in principle—can be beneficial in the analysis. One way to overcome future data inconsistencies is to standardise and harmonise the data, and international standardisation and harmonisation initiatives based on international standards and protocols are increasingly fostered for some environmental data such as land cover (Gregorio 2005; Herold *et al.* 2006); however, such initiatives are lacking for data regarding the social consequences of climate change, including migration. As for land cover data, such standardisation initiatives go beyond the influence of individual scientists and require internationally coordinated methods and efforts that include data agencies and policy-makers.

Ideally, studies that investigate environment-migration relationships are inspired by methods and data that stem from both migration studies and environmental studies. Beyond the methods already used in migration studies (see above) there are a couple of potentially beneficial approaches that, to our knowledge, have not been applied in migration studies. For example, climate models are frequently used for simulating ‘known’ historic climate to assess the forecasting system in predicting climate statistics (Taylor *et al.* 2011). Such so-called hind-casts may also provide important insights for migration modellers. Also, dating methods using quartz and feldspar optically stimulated luminescence—typically used for reconstructing landscape evolution processes during the past ~500.000 years (Preusser *et al.* 2008; Buylaert *et al.* 2012)—have shown to provide valuable information about historic migration (in addition to the already applied DNA analysis (Goebel *et al.* 2008)), such as when the genus ‘homo’ migrated out of Africa (Joordens *et al.* 2014). These examples glimpse the variety of methods originating from disciplines other than those typically applied in migration studies that may be beneficial for investigating the relationship between environmental change and migration.

A combination of various methods can help overcome the limitations of individual methods. Mixed-method research uses a research design that applies multiple methods (for example, quantitative and qualitative approaches) either independently or sequentially in order to understand certain phenomena such as environment-migration relationships (Tashakkori and Teddlie 2003). Combining several data collection approaches such as interviews and censuses can improve conclusions, also referred to as meta-inferences, which integrate findings from qualitative and quantitative approaches and are a major element of mixed-method research (Tashakkori and Teddlie 2008). Given the multidisciplinary nature of the environment-migration relationship we argue that mixed-method research is crucial. Some of the studies to which we have referred make use of an integrated approach, for example by combining remote sensing techniques with migration surveys or by expanding on-going surveys with additional questions regarding environmental change and migration. Although as noted such integrated research approaches are laudable, they usually require the expertise and experience of a relatively large inter-disciplinary team, which can make them expensive.

In addition, a combination of several approaches is often used to obtain improved data. As with the combination of methods, the respective strengths of various relevant datasets can be combined to overcome the limitations of each single dataset and to derive more robust and reliable data. In addition to improving the quality of the results, this can also contribute to the assessment of uncertainties related to the individual data sources. In general, all types of quantitative and qualitative data can be combined, including surveys and remote sensing data. To avoid possible data inconsistencies resulting from combining data, appropriate data handling must be applied, for example with respect to scaling and aggregation. However, such manipulations can potentially influence the characteristics of the data. As noted by Verburg *et al.* (2010), various methods exist for aggregating data, yielding different effects on the data. Whereas some aggregation methods lead to an overall loss of information, others can structurally change the representation of specific classes within the data. Therefore, the analyst must select the most appropriate aggregation method based on the data’s characteristics, which, along with its potential effects on the data should be documented thoroughly as it can provide a valuable basis for assessing uncertainty.

To advance the study of complex causal relationships between environmental change and migration, future research will need new approaches. Bilsborrow and Henry (2012) advocate looking beyond individual local field studies to the design of large-scale environment-migration relations projects in order to apply a consistent and comparable approach at several sites, which can be both time-intensive and costly. Therefore, despite their promising potential, comparable systematic studies are rare. An exception is the project “Where the Rain Falls,” a comparative study that systematically explores the interrelationships between rainfall variability, food and livelihood security, and migration at various research sites in Asia, Africa, and Latin America (Warner *et al*. 2012). A primary prerequisite for conducting a comparative study is clearly the standardisation of underlying data collection methods, assumptions, definitions, and data in order to avoid inconsistencies.

The number of individual case studies regarding environment-migration relationships is increasing, which necessitates the development of approaches designed to summarise case study findings and bring them to the attention of policy-makers. Systematic meta-analyses of case studies can be a valuable tool, as they facilitate the analysis of causal relationships identified in published case studies. Therefore, these studies can help answer the question of whether and how local, regional, and global specifics can be adequately represented and understood when assessing the environment-migration nexus. Literature-based meta-analyses have shown considerable promise for investigating the human-environment system. However, applications in the field of environmental change and migration are still lacking, and until recently, this paucity could be explained by the lack of data quantifying environmental change and migration at comparable scales (Piguet *et al*. 2011). Although current availability of data is far from adequate, some simple meta-analyses should be possible, given the increasing number of case studies (see Neumann and Hermans 2015). Taken as a whole combined case-study findings in standardised and/or summarised form may provide new insight into environmental change-related migration, for example with respect to regional trends in drought-related migration.
